# Molecular typing of clinical and environmental isolates of *Klebsiella pneumoniae* producing ESBLs by PFGE 

**DOI:** 10.22038/IJBMS.2022.58445.12981

**Published:** 2022-02

**Authors:** Mohammad Esmaeilnia, Mahmood Saffari, Somaye Rashki, Zeynab Marzhoseyni, Azad Khaledi, Gholam Abbas Moosavi, Fatemeh Atoof, Behrang Alani

**Affiliations:** 1 Department of Microbiology and Immunology, Faculty of Medicine, Kashan University of Medical Science, Kashan, Iran; 2 Infectious Diseases Research Center, Kashan University of Medical Science, Kashan, Iran; 3 Department of Vital Statistics and Epidemiology, School of Health, Kashan University of Medical Sciences, Kashan, Iran; 4 Department of Biostatistics, Health Faculty, Kashan University of Medical Sciences, Kashan, Iran; 5 Department of Applied Cell Sciences, Faculty of Medicine, Kashan University of Medical Sciences, Kashan, Iran

**Keywords:** Antibiotic resistance, ESBLs, ICU, Infection, Iran, Klebsiella pneumoniae, Medical equipment

## Abstract

**Objective(s):**

*Klebsiella pneumoniae *is the common cause of pneumonia in hospitalized patients, particularly in intensive care units (ICU). The infection can transfer by medical equipment such as mechanical ventilators. This study aimed to investigate the molecular typing of the extended-spectrum beta-lactamase-producing *K. pneumoniae* isolates recovered from Beheshti Hospital, Kashan, Iran.

**Materials and Methods:**

*K. pneumoniae* isolates producing ESBLs have been collected from the samples obtained from Shahid Beheshti hospital, Kashan, Iran. Antimicrobial susceptibility was determined using the Kirby Bauer disk diffusion method. The presence of ESBLs was evaluated using CLSI for ESBL screening by the double-disk diffusion method. Molecular typing was conducted by pulsed-field gel electrophoresis (PFGE). In total, 89 K. pneumoniae isolates were recovered, of which 47.1% were ESBL producers.

**Results:**

Results showed that all of the clinical and environmental isolates were resistant to ceftriaxone, meropenem, cefazolin, cefotaxime, cephalothin, and piperacillin-tazobactam. All isolates were grouped under four clusters (A-D). The major cluster was related to the C cluster with 22 isolates (19 clinical and 3 environmental). Seventy-two percent of isolates were from the ICU ward. There was no correlation between antibiotic resistance patterns and PFGE clusters (*P*=0.2).

**Conclusion:**

We observed a common molecular signature among both clinical and environmental *K. pneumoniae* isolates, indicating a similar genotype and likely a common origin for ESBL producer isolates found in different hospital wards. Therefore, hospitals need to implement an effective infection control system to decrease the spreading of ESBL strains within the hospitals and subsequently the transmission of the infection to patients.

## Introduction

*Klebsiella pneumoniae* is a Gram-negative opportunistic pathogen causing nosocomial and community-acquired infections (1). This microorganism is the most common cause of pneumonia among hospitalized patients, particularly in intensive care units (ICU), and can be transferred by medical equipment such as mechanical ventilators (2, 3). Over the past few decades, resistance of *K. pneumoniae* to antibiotics has become a major public health concern worldwide (4). It is noteworthy that the infections caused by multidrug-resistant (MDR) *K. pneumoniae* isolates have been closely related to increased morbidity and mortality, long hospital stay, and high healthcare costs (5). Among the most important mechanisms behind the increasing emergence and spread of antimicrobial-resistant isolates are the mobile genetic elements that carry virulence factors (6). These factors increase the colonization and establishment of the infection in the host’s body.

Antibiotic resistance is caused by various mechanisms; however, one of the most important mechanisms employed by Gram-negative bacteria against β-lactam antibiotics is producing extended-spectrum beta-lactamase (ESBL) enzymes (7). In recent years, the infections caused by ESBL-producing *K. pneumoniae* have been on the rise, creating a major health threat. It is worth mentioning that high resistance to antibiotics and disinfectants is one of the reasons for the long-term survival of this microorganism in different areas of hospitals and on contaminated medical equipment such as ventilators (8). These contaminated areas could act as sources for cross colonization and also as the major causes of hospital-based transmission of the microorganism, facilitating its circulation within the hospital environment, especially in the ICU ward (9, 10). So, obtaining data about the genetic signatures of environmental and clinical isolates of the bacterium and monitoring the sources of the infection in medical centers are essential (11, 12). This information can be useful in reducing the bacterial load, preventing the transmission of infectious agents, and decreasing the risk of infection (13). 

Molecular typing plays an important role in epidemiologic studies for determining genetic similarities between isolates and the sources of infections (14). This method also can be used to monitor the spread and genetic diversity of nosocomial pathogens such as *K. pneumonia *(15). A variety of methods have been used for molecular typing of *K. pneumonia*, such as pulsed-field gel electrophoresis (PFGE), enterobacterial repetitive intergenic consensus-polymerase chain reaction (ERIC-PCR), randomly amplified polymorphic DNA (RAPD), and multilocus sequence typing (MLST) (16, 17). Among these, PFGE has been considered the molecular gold standard for epidemiological studies because it shows the highest discriminatory power (18). This technique can differentiate clinical and environmental isolates based on changes in genomic fingerprinting patterns (19). Due to the importance of infection in ICU-admitted patients, there is a need for comprehensive research in this field(20). Therefore, in order to trace infection sources to help reduce hospital-acquired infections and control the spread of *K. pneumoniae* in the hospital environment, molecular typing of clinical and environmental isolates by the PFGE technique is an important procedure. This study aimed to characterize the antibiotic resistance profiles and clonal relatedness of environmental and clinical isolates of* K. pneumoniae.*

## Materials and Methods

This work was approved by the ethics committee of Kashan University of Medical Sciences (IR.KAUMS.MEDNT.REC.1397.103).

***Isolation and identification of bacteria***

Clinical samples (n=368) including tracheal aspirates, blood, ulcer, and urine were taken from 272 patients hospitalized in ICU and internal wards of Shahid Beheshti Hospital, Kashan, Iran from January 1^st^ to December 31, 2019. 

Ninety-six environmental samples were collected from the internal wall surface of mechanical ventilators and high-touch hospital surfaces such as bed rails, tabletops, and armrests in both wards. Then swab specimens were inoculated within two hours onto Tryptic Soy Broth (TSB), and the initial isolation was conducted on MacConkey (HiMedia Co, India) and blood agar (HiMedia Co, India). Microbiological (colony morphology and Gram staining) standard biochemical tests (such as citrate tests, urase tests, KIA tests, indole tests, MR tests, VP tests, and motility tests) were used to identify *K. pneumoniae.*

***Antimicrobial susceptibility testing***

The antibiotic susceptibility pattern of ESBL producing isolates was determined by the Kirby Bauer disk diffusion method according to CLSI instructions (21). The antibiotic disks used in this study were as follows; ceftriaxone (30 μg), meropenem (10 μg), cefazolin (30 μg), cefotaxime (30 μg), cephalothin (30 μg), piperacillin-tazobactam (100/10 μg), ampicillin (10 μg), aztreonam (30 μg), amikacin (30 μg), co-trimoxazole (25 μg), gentamicin (10 μg), ciprofloxacin (5μg), and tobramycin (10 μg). Antibiotic disks were provided by MAST Company (the UK). *Escherichia coli *ATCC 25922 was used as the reference strain.

***Phenotypic screening of extended-spectrum β-lactamase***

The presence of ESBLs was evaluated using CLSI criteria for screening these bacteria via the double-disk diffusion method (21). More than a 5-mm increase in the inhibition zone for ceftazidime-clavulanic acid disk when compared with ceftazidime disk alone was interpreted as phenotypic evidence of ESBLs production.

***Bacterial clonal relatedness***

ESBL producing isolates were typed by pulsed-field gel electrophoresis (PFGE, (Bio-Rad Laboratories, Hercules, CA, USA)). For this, chromosomal DNA was prepared using a methodology described by Kaufmann with some modifications (22). Chromosomal DNA from *K. pneumoniae* isolates was digested by XbaI restriction endonuclease (10 U/μl) under the following conditions: temperature of 14 *°C,* voltage of 6 V/cm, running time of 23 hr, and switch time of 1±30 sec. 

***Statistical analysis***

The BIONUMERICS software, version 7.5 (Bio-Rad, Hercules, CA, USA) was used to analyze macro-restriction fragment patterns, and dendrogram analysis was performed using the unweighted pair group method applying an arithmetic average (UPGMA). Isolates were grouped in clusters when there was a maximum difference of three bands in their macro-restriction patterns (i.e., a similarity level of about 80 %( (23). Categorical variables were abstracted as numbers (percentages). Pearson’s two-sided chi-square test or, if the expected count in any cell was less than required, Fisher’s exact test was used to compare these data.

## Results

***Bacterial isolates and epidemiological data***

In total, 89 *K. pneumoniae *isolates (80 clinical and 9 environmental) were recovered from the samples obtained from Shahid Beheshti hospital, Kashan, Iran from January 1^st^ to December 31, 2019. No *K. pneumoniae *isolate was recovered from blood and ulcer samples. One hundred and forty-four (53%), and 128(47%) of patients were male and female, respectively. The mean age of the patients was 49.1 ± 21.3 (range 16–87) years.

All isolates were detected and confirmed via microbiological and biochemical tests. ESBL-positive isolates were detected by confirmatory tests (i.e., the double-disk synergy test, DDST) according to CLSI 2019 guidelines. The results showed that 42 (47.1%) (Including 38 clinical and 4 environmental) isolates (out of a total of 89) were ESBL producers. Most clinical ESBL isolates (36/38, 94%) were obtained from the tracheal aspirates recovered from the patients hospitalized in the ICU (n=33, 86%) and internal ward (n=9, 23%). Also, environmental ESBL isolates were obtained from hospital surface areas (n=1, 25 %) and mechanical ventilators (n=3, 75 %,). 

***Findings of antimicrobial susceptibility testing***

**Figure 1:**
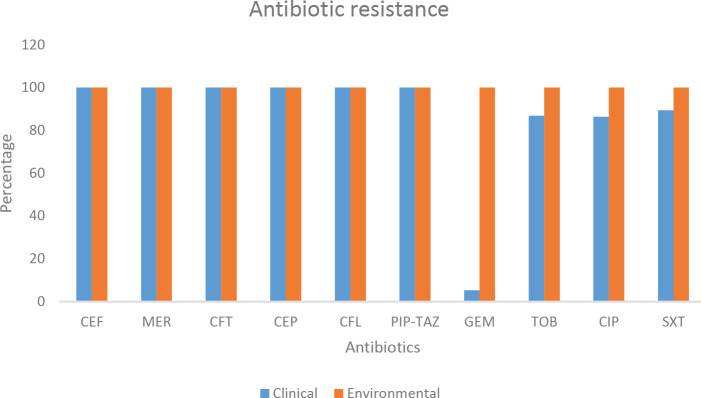
Percentage of antibiotic resistance in clinical and environmental *Klebsiella pneumoniae* isolates

The antibiotic resistance patterns of ESBL producing *K. pneumoniae* isolates against antimicrobial agents have been shown in Figure 1. Our results showed that all clinical and environmental isolates were resistant to ceftriaxone, meropenem, cefazolin, cefotaxime, cephalothin, and piperacillin-tazobactam. Among clinical isolates, the least antibiotic resistance was reported for Gentamicin 6 (15.79%). No statistically significant differences were found comparing antibiotic resistance patterns of different types of isolates recovered from different hospital wards.

***Genetic relatedness analysis***

**Figure 2:**
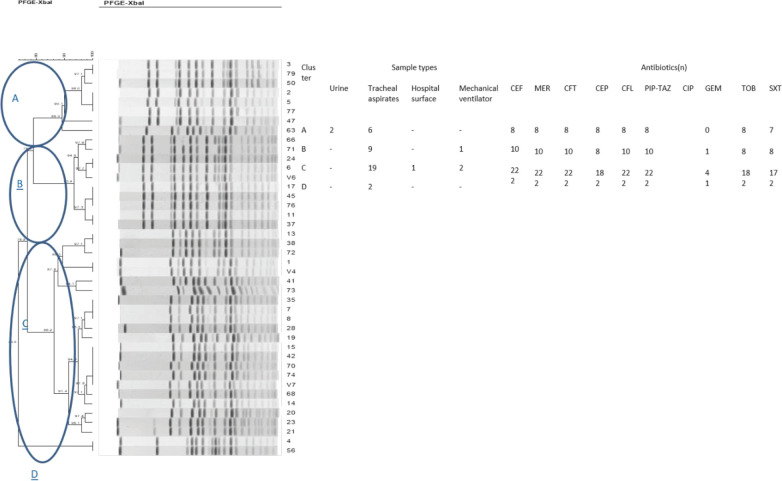
Dendrogram representing the PFGE profiles of 42 clinical and environmental *Klebsiella pneumoniae* isolates

**Figure 3.**
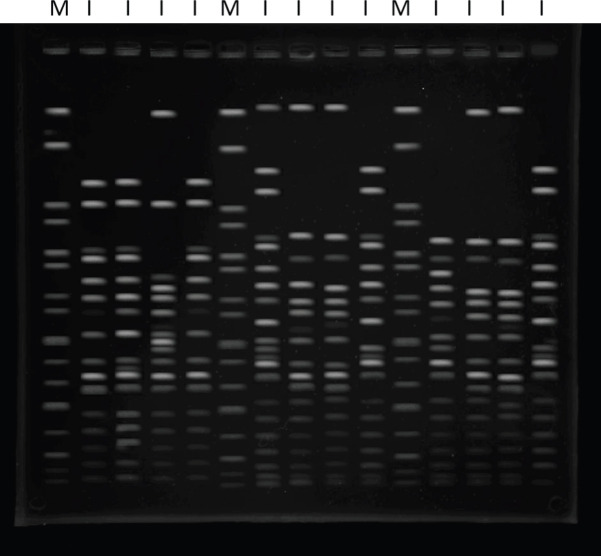
Xba1 macro-restriction patterns obtained for *Klebsiella pneumoniae* after the plug was lysed in a modified lysis buffer. H9812 strain of Salmonella serotype Braenderup was used as a molecular marker. M: marker, I: isolate

All 42 *ESBL*-positive *K*. *pneumoniae isolates *were genotyped by the PFGE technique. Based on the number of bands observed on the dendrogram (Figures 2 and 3), the isolates were grouped in four clusters (A-D). Also, the findings of PFGE analysis showed a genotypic similarity of 80% among the four clusters. Regarding the distribution of 42 isolates among these clusters, the majority of them were categorized under cluster C (22 isolates, 19 clinical and 3 environmental), 72% of which were from the ICU. In addition, most of the isolates obtained from ventilators and tracheal aspirates were placed under cluster C (Table 1). On the other hand, the clusters A, B, and D contained 8 (19%), 10 (24%), and 2 (5%) isolates, respectively. Most of the isolates (cluster C) were recovered from tracheal aspirates and ventilators, respectively. Under cluster B, there were 10 isolates, most of which were retrieved from tracheal aspirates and showed a similar antimicrobial susceptibility pattern. Also, among these, one identified environmental isolate (from the internal ward) showed a band pattern similar to those isolated from the ICU. Six out of eight isolates in cluster A belonged to tracheal aspirates that had a genetic similarity of over 90%. The other two isolates (from the internal ward and ICU) were retrieved from urine samples and showed a completely similar genetic signature. The cluster D contained two isolates, both recovered from the ICU and tracheal aspirates. These had exactly the same antimicrobial susceptibility profile. The similarity between the PFGE patterns of environmental and clinical isolates indicated circulation of *K*. *pneumoniae *isolates in the ICU and the role of environmental transmission in the spread of the infection. There were no correlations between antibiotic resistance patterns and PFGE clusters.

## Discussion

In the present study, 47.1% of the isolates were ESBL producers, of which 90.47% were clinical isolates and the remaining were environmental. In addition, the findings of antibiotic susceptibility testing showed that all ESBL isolates were resistant to cephalosporins, meropenem, and piperacillin-tazobactam, suggesting an urgent need for revising and modifying the pattern of antibiotic consumption because, in the near future, various pan drug resistance (PDR) strains of this microorganism will appear, leaving no antibiotics for treatment. Recent studies have shown that beta-lactamase enzymes play an important role in the development and transmission of antibiotic resistance, so, ESBL strains impose health threats on hospitalized patients and individuals with weak immune systems. For example, in a study conducted by Hashemizadeh *et al*., 86.6% of isolates produced broad-spectrum beta-lactamase enzymes (18). In another study conducted by Langarizadeh *et al*. in Tabriz, Iran on *K. pneumoniae* isolates, 98.61% of them were MDR strains, and chloramphenicol, norfloxacin, amikacin, ciprofloxacin, and imipenem had the greatest effects on them (24). Japaninejad *et al*., in a study in Tehran, Iran in 2014, also reported a high percentage of *K. pneumoniae* isolates with high antibiotic resistance (25), which is consistent with the findings of the present study. Similarly, the studies conducted in western Iran, China, and Egypt, reported the prevalence of resistance to carbapenems 50% and 60%, respectively (26-28), indicating a lower rate than the one reported in the present study (28). 

In order to study the epidemiological aspects of such infections and identify their sources, molecular methods, especially PFGE, are useful (29). Now, this technique is widely accepted as the most powerful and commonly used molecular tool for epidemiological studies on bacterial strains, including *K. pneumoniae*, due to its ability to differentiate various strains, as well as good reproducibility and easy interpretation. Although it is believed that determining the nucleic acid sequence of genes is the best way to divulge epidemiological relationships between bacterial strains, the PFGE method is referred to as the gold standard technique today. In the present study, according to the results of PFGE analysis, four different genetic patterns (AD) were identified with the highest and lowest numbers of isolates belonging to clusters C and D, respectively. In cluster C (n = 22), most of the isolates were recovered from tracheal aspirates and ventilators. These isolates can be horizontally transmitted between patients and staff through hospital equipment such as ventilators, so a ventilator can be considered as a common environmental cause of infection among patients. Therefore, if not enough attention is paid to control these strains, we may face a high rate of possible epidemics caused by these strains in the future, which is a serious warning for physicians and the infection control team. 

Under cluster B, there were 10 isolates, most of which were retrieved from tracheal aspirates and showed a similar antimicrobial susceptibility pattern. Also, among these, one identified environmental isolate (from the internal ward) showed a band pattern similar to those isolated from the ICU, so it is possible that it was recently released from the ICU to the internal ward, which could alarm widespread dissemination. It could have just been transferred from the ICU to the internal department; of course, the opposite may have happened as well. In addition, in the mentioned cluster, an isolate was recovered from a ventilator, indicating the importance of this apparatus in the transference of this infection among different wards of hospitals. Six out of eight isolates in cluster A belonged to tracheal aspirates that had a genetic similarity of over 90%. The other two isolates (from the internal ward and ICU) were retrieved from urine samples and showed a completely similar genetic signature. According to this, it can be concluded that there is a possibility for either endogenous (from the person himself/herself) or exogenous (through the bathroom) transfer. A noteworthy point in this cluster was the existence of isolates from the ICU and internal ward with similar band patterns. The cluster D contained two isolates, both recovered from the ICU and tracheal aspirates. These had exactly the same antimicrobial susceptibility profile, which could indicate that the bacteria had recently entered the ward and were probably community-acquired, brought to the hospital by patients or clients. Furthermore, the genetic persistence of the two isolates had probably a role in the survival, colonization, and spread of the bacteria. 

Due to the importance of studying the molecular epidemiology of *K. pneumoniae*, various studies have been conducted around the world. In a study conducted in Canada from 2008 to 2011 on 26 isolates of carbapenemase-positive *K. pneumoniae*, PFGE analysis revealed three clusters, among which the C1 cluster with 23 isolates and a genetic similarity above 80% was the dominant cluster (30); these results were similar to those of the present study. In another study conducted in Bosnia and Herzegovina in 2007 on 57 ESBL *K. pneumonia* isolates collected from the patients admitted to different wards of a reference hospital, PFGE analysis showed four clusters, of which cluster A with 35 isolates was the dominant category (31). However, in the recent study, compared with our study, there was a great diversity in genomic patterns regarding the number of samples, which was due to dispersion of samples and the different origins of the strains. In a study conducted in Egypt on the *K. pneumoniae* isolates recovered from clinical and environmental samples, PFGE analysis showed high genetic diversity among clusters (32). Also, in another study conducted by Pirayeh *et al*., 11 clusters were detected based on PFGE genotyping, which was different from those observed in the present study, probably reflecting the different sources (hospital wards) of strains (33). In another study, 35 isolates of ESBL producing *K. pneumoniae* were collected from the patients admitted to different hospital wards, of which more than half of the isolates were from urine samples, and the rest were grown from lung secretions, wounds, blood, sputum, and unknown sources. The visual comparison of PFGE derived band patterns showed six clusters with 70% similarity and 21 clusters with 85% similarity (34). In a study conducted on 31 CTX-M-producing *K. pneumoniae *isolates from urine, sputum, wounds, blood, tracheal aspirates, CSF, etc., PFGE analysis showed 26 different patterns (35). Also, among 54 *K. pneumoniae* strains isolated from urine, lung, wound, blood, sputum, and other samples, PFGE analysis showed 22 clusters (A-W) with 70% similarity and 42 clusters with 85% similarity (36). In these studies, the genetic diversity among isolates was high; one reason for this could be the diversity of sample sources, and because our samples were not from diverse sources, our findings were somehow different from the results of some of the previous studies. In line with our research, however, there are also studies reporting a low genetic diversity. For example, a study in Tanzania between 2009 and 2010 was conducted on 92 ESBL-producer *K. pneumoniae* isolates recovered from blood, respiratory, urine, and pus samples. Most blood cultures were from the neonatal and NICU units, and 13 patterns were obtained following PFGE analysis (37). 

CEF: Ceftriaxone, MER: Meropenem, CFT: Cefotaxime, CEP: Cephazoline, CFL: Cefalotin, PIP-TAZ: Piperacillin/tazobactam, CIP: Ciprofloxacin, TOB: Tobramycin, SXT: Sulfamethoxazole and trimethoprim, GEM: Gentamicin

CEF: Ceftriaxone, MER: Meropenem, CFT: Cefotaxime, CEP: Cephazoline, CFL: Cefalotin, PIP-TAZ: Piperacillin/tazobactam, CIP: Ciprofloxacin, TOB:Tobramycin, SXT: Sulfamethoxazole and trimethoprim, GEM: Gentamicin

**Table 1:** Distribution of 42 clinical and environmental *Klebsiella pneumoniae* isolates among the four clusters (A-D).

**Cluster**	Sample types				
	Urine	Tracheal aspirates	Hospital surface	Mechanical ventilator	Total N (%)
A B C D	2 - - -	6 9 19 2	- - 1 -	- 1 2 -	8(19) 10(24) 22(52) 2(5)

## Conclusion

Our study showed that most isolates were recovered from tracheal aspirates and ventilators. These isolates can be transferred horizontally between patients and medical staff through hospital equipment such as ventilators, so such apparatus can be considered as common environmental causes of the infection. Also, we observed the strains isolated from the ICU and internal ward, which showed a similar band pattern. So, the presence of such isolates with similar genotypes may imply a common origin and reflect the dissemination of ESBL isolates across hospital wards. Therefore, hospitals need to implement an effective infection control system to decrease the spreading of MDR strains in the hospital environment and their transmission to patients.

## Authors’ Contributions

ME and MS Provided the study conception and design; SR Performed acquisition of data; ZM and AK Wrote the manuscript in consultation with GAM, FA, BA; Critical revision. All authors have read and agreed with all contents included in the present article.

## Conflicts of Interest

The authors express no conflicts of interest. 
